# Electrospun 5-Chloro-7-iodo-8-hydroxyquinoline (Clioquinol)-Containing Poly(3-hydroxybutyrate)/Polyvinylpyrrolidone Antifungal Materials Prospective as Active Dressings against Esca

**DOI:** 10.3390/polym14030367

**Published:** 2022-01-18

**Authors:** Milena Ignatova, Nasko Nachev, Mariya Spasova, Nevena Manolova, Iliya Rashkov, Mladen Naydenov

**Affiliations:** 1 Laboratory of Bioactive Polymers, Institute of Polymers, Bulgarian Academy of Sciences, Acad. G. Bonchev St, Bl. 103A, BG-1113 Sofia, Bulgaria; nachev_n@polymer.bas.bg (N.N.); manolova@polymer.bas.bg (N.M.); rashkov@polymer.bas.bg (I.R.); 2 Department of Microbiology, Agricultural University, BG-4000 Plovdiv, Bulgaria; mladen@au-plovdiv.bg

**Keywords:** 5-chloro-7-iodo-8-hydroxyquinoline (clioquinol), poly(3-hydroxybutyrate), polyvinylpyrrolidone, electrospinning, electrospraying, antifungal activity, *Phaeoacremonium aleophilum*, *Phaeomoniella chlamydospora*

## Abstract

Esca is a grapevine disease known for centuries which pertains to the group of so-called vine trunk diseases. *Phaeomoniella chlamydospora (P. chlamydospora)* and *Phaeoacremonium aleophilum (P. aleophilum)* are the two main fungal pathogens associated with esca. Novel fibrous materials with antifungal properties based on poly(3-hydroxybutyrate) (PHB), polyvinylpyrrolidone (PVP) and 5-chloro-7-iodo-8-hydroxyquinoline (clioquinol, CQ) were developed. One-pot electrospinning (“*in*” strategy) or electrospinning in conjunction with electrospraying (“*on*” strategy) were applied to obtain the materials. The materials’ morphology and their surface chemical composition were examined using scanning electron microscopy (SEM), X-ray photoelectron spectroscopy (XPS) and attenuated total reflection Fourier transform infrared spectroscopy (ATR-FTIR). CQ incorporated in the bulk of the fibers or in PVP particles deposited on the fibers was in the amorphous phase, which was confirmed by differential scanning calorimetry (DSC) and X-ray diffraction analysis (XRD). The in vitro release of CQ depended on the composition of the electrospun materials and on their design. The performed microbiological screening revealed that, unlike the non-loaded mats, the fibrous mats loaded with CQ were effective in inhibiting the growth of the pathogenic *P. chlamydospora* and *P. aleophilum* fungi. Therefore, the created materials are promising as active dressings for grapevine protection against esca.

## 1. Introduction

Esca is a grape disease known since antiquity which pertains to the so-called grapevine trunk diseases leading to the fading of leaves and limbs and to entire trees wilting [1,2,3]. The two main fungal pathogens that have been associated with esca are *Phaeomoniella chlamydosporum* (*P. chlamydospora*) and *Phaeoacremonium aleophilum* (*P. aleophilum*) [4,5]. Mostly, the entire vineyard must be replanted, as fungal spores are airborne and infect other vines mainly through wounds caused during pruning. Esca induces huge economic losses by reducing the yield of the vine and its longevity [6]. Up to now, sodium arsenite, a popular fungicide, has been used as an agent to effectively combat against esca disease. Recently, this fungicide has been recognized as carcinogenic and at present has been banned [7]. Thus, in practice, it turns out that there are no effective approaches to fighting against esca. Currently, solely preventive approaches are used [8]. Therefore, there is a need to develop novel active substances and effective materials which are non-toxic and efficient in protecting the vine plants against esca.

Electrospinning is a feasible and beneficial method for the fabrication of fibers having diameters in the micrometer or nanometer range using an external electric field applied to a polymer-containing solution or melt [9,10]. Recently, fibrous polymeric materials obtained by electrospinning have aroused considerable interest due to their superb properties, such as a large specific surface area, high porosity and three-dimensional structure. Therefore, they are prospective candidates for a variety of applications: in medicine and pharmacy as wound dressings [11]; drug delivery systems [12]; as tissue engineering scaffolds [13]; as filtration membranes; in cosmetics; as protective clothing; the design of nanosensors; in electronics; and in agriculture [14].

At the present time, there are a limited number of publications reporting the fabrication of electrospun materials for grapevine protection against esca disease. Sett et al. have reported the creation of fibers based on a biodegradable rayon membrane onto which nanofibers from soy protein, polyvinyl alcohol and polycaprolactone were electrospun [15]. These materials were designed for physically blocking the infiltration of pathogenic spores. Unfortunately, physical hampering was found to be unsatisfactory in combating esca disease [16]. Therefore, electrospun poly(butyleneadipate-*co*-terephthalate) materials containing a polymer additive with antifungal activity polyhexamethylene guanidine have been proposed for active protection against penetration of *P. chlamydospora* spores causing esca [16]. However, the authors have underlined the necessity of a more appropriate selection of polymers and more effective antifungal additives. 

Recently, it has been reported by some of us that eco-friendly fibrous material composed of poly(3-hydroxybutyrate) (PHB), TiO_2_-anatase nanoparticles and chitosan with low molecular weight prepared using simultaneous electrospinning and electrospraying displayed good antifungal activity against *P. chlamydospora* [17].

The 8-hydroxyquinoline derivatives exhibit diverse biological activities: antitumor, antiviral, neuroprotective, antimicrobial, and antifungal [18,19,20]. They also have low toxicity to humans. Some of us have reported the successful encapsulation of 8-hydroxyquinolines in electrospun materials based on cellulose derivative and poly(ethylene glycol) [21] and polylactide [22] to obtain materials that provide effective protection by impeding the entrance and growth of fungal spores on vine plants.

In this study, 5-chloro-7-iodo-8-hydroxyquinoline (CQ) was selected as a model compound belonging to the group of 8-hydroxyquinoline. Currently, it is used as an active compound in topical medications to treatment diverse skin infections. CQ has good antimicrobial [23,24], antifungal [24,25] and antitumor activity [26], and has a potential beneficial effect in the treatment of Parkinson’s, Alzheimer’s and Huntington’s diseases [27,28,29]. When incorporated into electrospun polymeric materials, this compound can impart beneficial biological properties.

Among the polymers suitable for applications in agriculture, aliphatic polyesters such as polyhydroxyalkanoates, poly(lactic acid) and their copolymers are of considerable interest. PHB is particularly promising. It is a thermoplastic, hydrophobic polymer which possesses physical properties very close to polypropylene. In addition, PHB is biodegradable, non-toxic, and UV resistant [30,31,32]. PHB can be easily electrospun and the obtained fibrous materials have good mechanical properties.

Polyvinylpyrrolidone (PVP) was chosen for use in the fibrous materials applied for agricultural purposes because it is a non-toxic, biocompatible, biodegradable, and pH-stable nonionogenic water-soluble polymer [33,34]. It has been proved that fibrous materials based on PVP are suitable for the delivery of poorly water-soluble biologically active compounds, because PVP diminish crystal formation in the loaded biologically active compounds and increases their dissolution rate in aqueous medium [35,36,37].

Until now, there have been no literature reports presenting the preparation of fibrous materials based on PHB and PVP containing CQ.

In this study, we have studied the preparation of novel antifungal materials based on PHB and PVP containing CQ of different designs. Two types of CQ-containing fibrous materials were fabricated using one-step electrospinning (type “*in*”) or simultaneous electrospinning and electrospraying (type “*on*”). Thus, the obtained mats were fully morphologically, physico-chemically and structurally characterized. In view of the potential use of the CQ-containing fibrous materials as active dressings for grapevine protection against esca disease, their antifungal activity against *P. chlamydospora* and *P. aleophilum* fungal species was evaluated.

## 2. Materials and Methods

### 2.1. Materials 

**Scheme 1:**
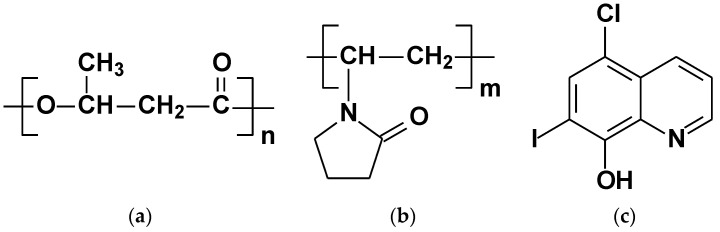
Structure of (**a**) the poly(3-hydroxybutyrate) (PHB), (**b**) polyvinylpyrrolidone (PVP) and (**c**) 5-chloro-7-iodo-8-hydroxyquinoline (clioquinol) (CQ) used in the present study.

In the present study the following polymers were used: poly(3-hydroxybutyrate) (Scheme 1a) (PHB, 330,000 g.mol^−1^, Biomer, Schwalbach, Germany) and polyvinylpyrrolidone (Scheme 1b) (PVP K25; Fluka, Buchs, Switzerland) with Mr = 24,000 g.mol^−1^. 5-chloro-7-iodo-8-hydroxyquinoline (clioquinol) (CQ) (Scheme 1c) was purchased from Sigma-Aldrich, Buchs, Switzerland. *N,N*-Dimethylformamide (DMF) (Merck, Darmstadt, Germany), chloroform (Merck, Darmstadt, Germany), acetone (Sigma-Aldrich, Buchs, Switzerland) and dimethyl sulfoxide (Merck, Darmstadt, Germany) were of an analytical grade of purity. The microbiological growth media was potato dextrose agar medium (Merck, Darmstadt, Germany).

### 2.2. Fabrication of the Mats

#### 2.2.1. Fabrication of PVP,CQinPHB Mats by Electrospinning

PHB spinning solution with a polymer concentration of 10 wt.% in CHCl_3_/DMF (4/1 *v*/*v*) was prepared for the fabrication of the PHB fibers. For that purpose, heating at 60 °C using a reflux condenser was used. PHB/PVP fibers will be further denoted as PVP*in*PHB, where a PHB/PVP weight ratio is 90/10 at a total polymer concentration of 10 wt.% (CHCl_3_/DMF (4/1 *v*/*v*)). The fibrous mats containing CQ in the bulk will be further indicated as PVP,CQ*in*PHB. For their fabrication, mixed solutions of CQ and PHB/PVP (90/10 *w*/*w*) in CHCl_3_/DMF (4/1 *v*/*v*) at a total polymer concentration of 10 wt.% and CQ concentration of 10% (% in weight to PHB/PVP content) were used.

The prepared solutions were loaded separately into plastic syringe (5 mL) placed horizontally in syringe pump [NE-300 Just Infusion^TM^ Syringe Pump (New Era Pump Systems Inc., Farmingdale, NY, USA)] and were delivered at a constant feed rate of 2 mL/h. The used tip-to-collector distance was 25 cm at a constant collector rotation speed of 1400 rpm. The used voltage was 25 kV ensured by a custom-made high voltage power supply. Finally, the obtained fibrous mats were additionally dried under a reduced pressure at 30 °C for 8 h to remove any residual solvents.

The dynamic viscosity of the spinning solutions was measured using a Bookfield DV-II+ programmable viscometer (Middleboro, MA, USA) for cone/plate option equipped with a sample thermostat cup and a cone spindle, at 25 ± 0.1 °C.

#### 2.2.2. Fabrication of PVP,CQonPHB Mats by Electrospinning in Conjunction with Electrospraying

The electrospun PHB fibrous materials decorated with PVP particles containing CQ were further denoted as PVP,CQ*on*PHB. These materials were fabricated by simultaneous electrospinning and electrospraying. PVP,CQ*on*PHB mats were prepared using a PHB spinning solution with concentration 10 wt.% in CHCl_3_/DMF (4/1 *v*/*v*) for electrospinning and a mixed solution of CQ and PVP in ethanol/DMSO (4/1 *v*/*v*) at a polymer concentration of 4 wt.% and CQ concentration of 10 wt.% (with respect to the PVP weight) for electrospraying. The prepared PHB and PVP,CQ solutions were loaded in two separate syringes. The syringes were placed in two infusion pumps (NE-300 Just Infusion^TM^ Syringe Pump from New Era Pump Systems Inc. (Farmingdale, NY, USA)), located on both sides of the collector at an angle of 180°. The PHB solution was delivered at a controlled feed rate of 2.0 mL/h, and that of PVP,CQ at 3 mL/h. The tip-to-collector distance was 25 cm for the electrospinning of the PHB solution and 15 cm for the electrospraying of the PVP,CQ solution. The electrospinning and electrospraying were carried out at a voltage of 25 kV using a common high-voltage power supply. The fibers decorated with particles were collected onto aluminum foil fixed to a grounded drum rotating with a speed of 1400 rpm. PVP,CQ*on*PHB mats were placed under reduced pressure at 25 °C to remove solvent residues.

### 2.3. Characterization of the Mats

The detailed morphological analysis of the fabricated fibrous materials was performed using scanning electron microscopy (SEM). Prior to observation all the samples were vacuum-coated with gold for 60 s in Jeol JFC-1200 fine coater and were analyzed by Jeol JSM-5510 (JEOL Co. Ltd., Tokyo, Japan). The mean fiber diameter and the standard deviation were assessed by measuring at least 30 fibers from SEM images using Image J software [38].

X-ray diffraction analysis (XRD) was performed to assess the crystalline structure of the fabricated fibrous materials. D8 Bruker Advance powder diffractometer (Bruker, Billerica, MA, USA) with a filtered CuKα radiation source and a luminescent detector was used to record the XRD patterns in the range 2θ range from 5° to 60° (step of 0.02° and counting time of 1 s/step).

To characterize the fabricated fibrous materials, attenuated total reflection Fourier transform infrared (ATR-FTIR) spectroscopy was carried out. The spectra were recorded on IRAffinity-1 spectrophotometer (Shimadzu Co., Kyoto, Japan) equipped with an ATR attachment with diamond crystal. The spectra were scanned in the mid-IR range from 600 to 4000 cm^−1^. The resolution of the spectra was 4 cm^−1^, and the scans were repeated 50 times. The spectra were corrected for H_2_O and CO_2_ using an IRsolution internal software.

X-ray Photoelectron Spectroscopy (XPS) was performed to investigate the surface chemical composition of the obtained fibrous materials. ESCALAB-MkII (ThermoFisher Scientific, Waltham, MA, USA) spectrometer using Mg Kα excitation equipped with ultrahigh-vacuum (UHV) chamber was used.

Differential scanning calorimetry (DSC) was carried out on DSC Q200 equipment (TA Instruments, New Castle, DE, USA) in the temperature range of 0 to 220 °C with a heating rate of 10 °C/min under nitrogen flow. The melting temperatures (T_m_) and fusion enthalpies ($\Delta H_{m}$) were obtained from the DSC endotherms (first heating run). Following Equation (1) was used to determine the crystallinity degree of PHB ($\chi_{c}^{\mathrm{PHB}}$,%) into the fibers:

(1) $$\chi_{c}^{\mathrm{PHB}},\%=\left[\frac{\Delta H_{m}^{\mathrm{PHB}}}{\Delta H_{m}^{\mathrm{PHB},0}\times W^{\mathrm{PHB}}}\right]\times 100$$

where $\Delta H_{m}^{0}$ was the fusion enthalpy of 100% crystalline PHB ($\Delta H_{m}^{\mathrm{PHB},0}$ = 146.6 J/g [39]); $\Delta H_{m}$ was the melting enthalpy of PHB during the heating cycle; $W^{\mathrm{PHB}}$ was the mass fraction of PHB in the materials. 

Static water contact angle of the fibrous materials was assessed using an Easy Drop DSA20E Krűss GmbH apparatus (Hamburg, Germany). A sessile drop of deionized water (volume-10 μL) was deposited onto the surface of the fibrous samples and the average value of the contact angle was determined 10 min after droplet deposition by computer analysis. 20 measurements for each sample were performed.

### 2.4. In Vitro CQ Release from the Fibrous Mats

CQ release was studied in vitro at temperature of 25 °C in acetate buffer medium (CH_3_COONa/CH_3_COOH) containing Tween 80 (acetate buffer/Tween 80 = 99/1 *v*/*v*), at pH 3.6 and ionic strength of 0.1. 60 mg of CQ-containing mat was immersed in 100 mL of buffer solution under stirring at 100 rpm in a thermally controlled shaking water bath (JULABO SW23, Allentown, PA, USA). At definite time intervals, aliquots were withdrawn and the amount of CQ released was determined by a DU 800 UV–vis spectrophotometer (Beckman Coulter, Brea, CA, USA) at a wavelength of 315 nm. The withdrawn volumes were replaced with fresh buffer solution. Calibration curves (correlation coefficient R = 0.999) were used to determine the released CQ. The release was repeated three times and the data were averaged.

### 2.5. In Vitro Antifungal Assay

In the present study the fungi *P. chlamydospora* CBS 239.74 and *P. aleophilum* CBS 631.94 purchased from Westerdijk Fungal Biodiversity Institute (Utrecht, Netherlands) were used. The minimum inhibitory concentration (MIC) of CQ against the two used fungi was assessed. A preculture of the different fungi was grown for seven days on potato dextrose agar medium (PDA, Merck, Darmstadt, Germany) at 28 °C. Spores were harvested by disposable L-shaped spreaders after flooding the culture with sterile water. The tested CQ was dissolved in DMSO at an initial concentration of 0.010 g/mL. Additional solutions were obtained by the serial dilution method. A total of 10 µL of each dilution was added to 990 µL of sterile potato dextrose broth in Eppendorf tubes. The resulting mixture was inoculated with 50 mL of spore suspension and incubated at 28 °C for 96 h. The detection of the fungal growth was performed using microscopic observation.

The antifungal activity of the samples from PHB, PVP*in*PHB (blank control), PVP,CQ*in*PHB and PVP,CQ*on*PHB was assessed by the disk-diffusion method described by Falcón-Piñeiro et al. [40] with some modifications. The effect on mycelial growth of *P. chlamydospora* CBS 239.74 and *P. aleophilum* CBS 631.94 was determined as follows: Discs with diameters of 17 mm were cut from all the fabricated fibrous materials. Then, they were placed in the Petri dishes (with diameters of 90 mm) previously inoculated with 0.1 mL of suspension of fungi culture (1 × 10^5^ cells/mL). The Petri dishes with fibrous samples were incubated for 96 h at 28 °C and finally the inhibition zones around each disk were determined.

## 3. Results and Discussion

### 3.1. Fabrication and Characterization of Fibrous Materials

The combining of the beneficial properties of the aliphatic polyester PHB and of water-soluble polymer PVP with the antifungal properties of CQ is a favorable strategy for the fabrication of novel fibrous materials appropriate for diverse applications in agriculture.

**Figure 1:**
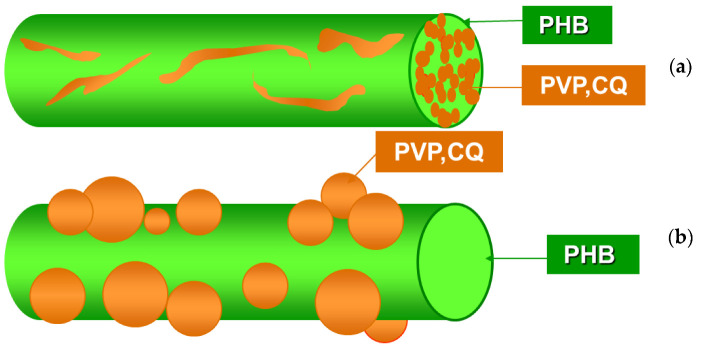
Schematic representation of fibers: (**a**) PVP*in*PHB fiber loaded with CQ in the bulk (PVP,CQ*in*PHB) and (**b**) PHB fiber decorated with PVP,CQ particles on the surface (PVP,CQ*on*PHB).

In this study, for the fabrication of fibrous materials from PHB and PVP containing CQ of diverse design, two different approaches were developed: one-step electrospinning of fibers from a solution of PHB, PVP and CQ (“*in*” strategy, Figure 1a) and electrospinning of a PHB solution in conjunction with electrospraying of a PVP,CQ solution (“*on*” strategy, Figure 1b).

**Figure 2:**
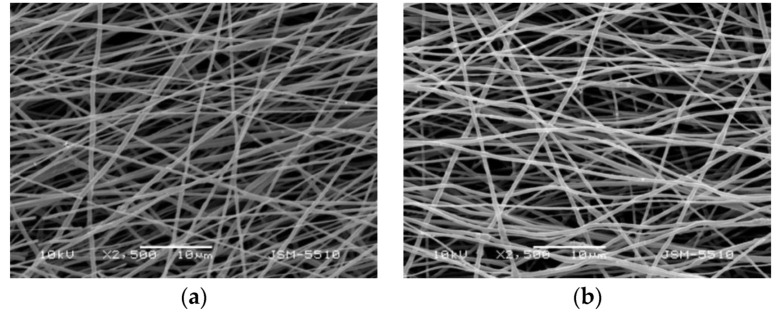

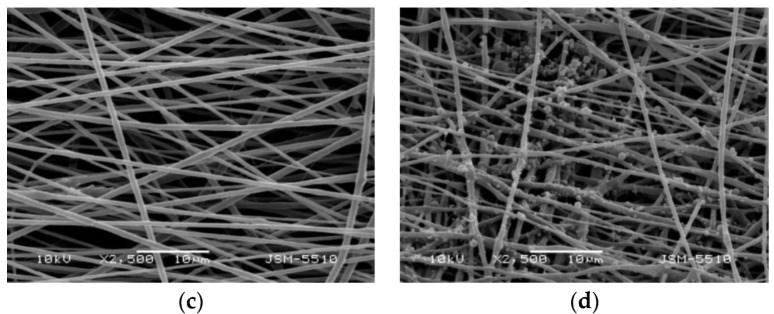
SEM micrographs of the fibers of: (**a**) PVP*in*PHB; (**b**) PVP,CQ*in*PHB; (**c**) PHB; and (**d**) PVP,CQ*on*PHB. Magnification ×2500.

The electrospinning of solutions of PHB, PVP and CQ in a CHCl_3_/DMF solvent system (4/1 *v*/*v*) at a total polymer concentration of 10 wt.% led to the formation of cylindrical and defect-free fibers (Figure 2b). PHB (Figure 2c) and PVP*in*PHB fibers (90:10 *w*/*w*) (Figure 2a) were also obtained using the selected conditions. The mean diameter of the PHB fibers was 760 ± 200 nm. The addition of PVP to the PHB solutions imparted a decrease in the average fiber diameters to 480 ± 110 nm (Figure 2a,c). This effect is most likely due to a decrease in the viscosity of the PHB solution from 180 cP to 110 cP with the addition of PVP. When CQ (10 wt.%) was added to the PHB/PVP solution, the average fiber diameter changed only negligible (Figure 2b)—from 480 ± 110 nm for the PVP*in*PHB mat to 470 ± 110 for the PVP,CQ*in*PHB mat. This might be attributed to the slight decrease in the viscosity of the PHB/PVP solution from 110 cP to 100 cP on adding of CQ to the solution.

PHB fibers decorated with PVP,CQ particles were obtained by performing simultaneous electrospinning and electrospraying. SEM micrographs of PVP,CQ*on*PHB mats are presented in Figure 2d. It is evident that the particles of PVP,CQ deposited on the PHB fibers had spherical shape. The average particle size for the PVP,CQ*on*PHB mats was 490 ± 150 nm (Figure 2d).

**Figure 3:**
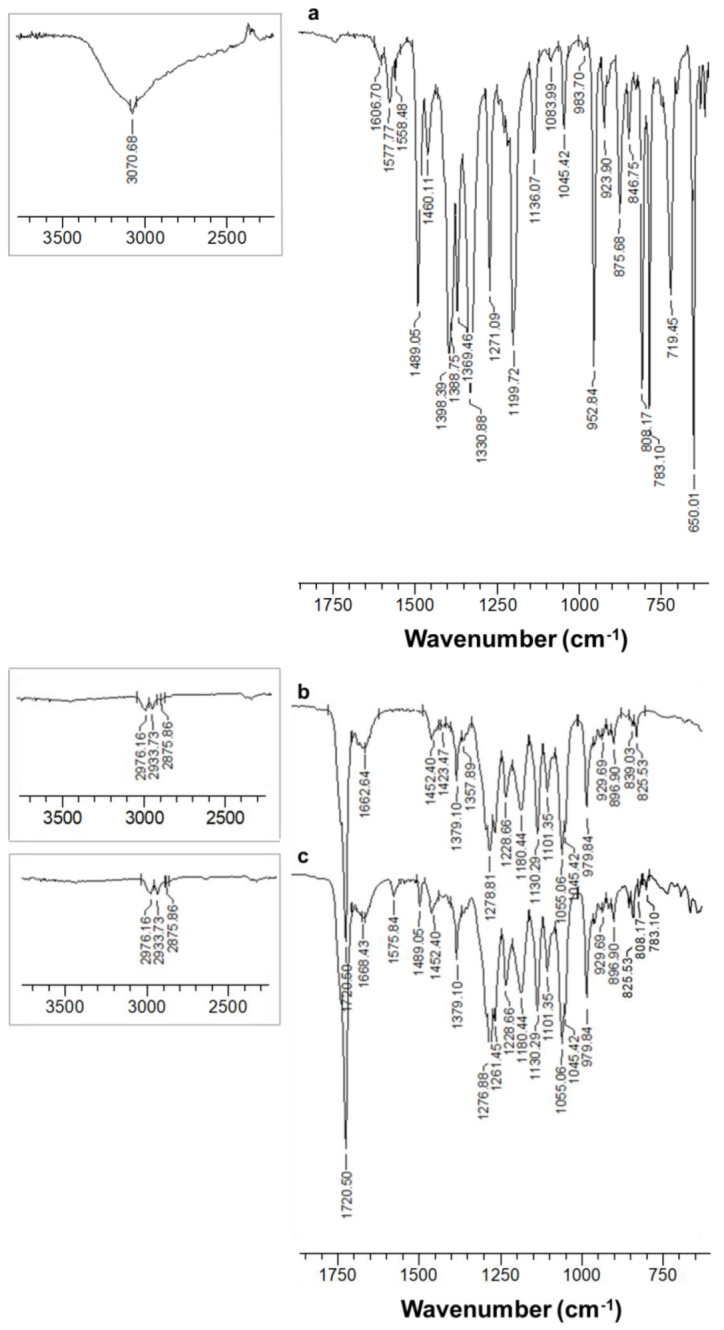

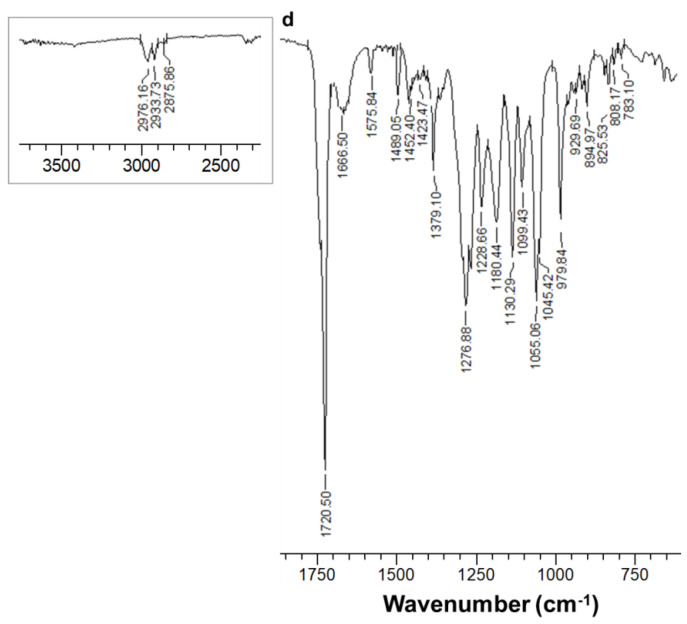
ATR-FTIR spectra of: (**a**) CQ; (**b**) PVP*in*PHB mat; (**c**) PVP,CQ*in*PHB mat; and (**d**) PVP,CQ*on*PHB mat in the range from 1750 to 750 cm^−1^ and from 3500 to 2500 cm^−1^ (inset).

For confirmation of the chemical structure of the CQ-containing fibrous material, ATR-FTIR spectroscopy was carried out (Figure 3). In the ATR-FTIR spectra of PVP*in*PHB mats, in addition to the absorption characteristic bands of PHB (1721 cm^−1^—ν_C=O_ [35]; 1279, 1229, 1180 cm^−1^—ν_as C–O–C_ in the crystalline and amorphous phases [41,42]), a band appeared at 1663 cm^−1^, characteristic for ν_C=O_ vibrations from the PVP (Figure 3b and Supplementary Material, Figure S1). In the case of the PVP,CQ*in*PHB mat, the band for C=O stretching vibrations of PVP was shifted towards the higher wavenumbers to 1668 cm^−1^ (by 5 cm^−1^) compared to the spectrum of the neat PVP*in*PHB mat (1663 cm^−1^) (Figure 3b,c). This is in conformity with previous reports for other PVP-based fibrous materials loaded with natural phenolic compounds [43,44]. This shift is most likely due to an intermolecular interaction based on hydrogen bonds between PVP and CQ. In the case of CQ-containing fibers, the appearance of characteristic bands for CQ [45] (stretching vibration bands of the CQ ring (1576 cm^−1^ and 1489 cm^−1^) and bands at 808 cm^−1^ for the benzene ring (γ_Ar-H_, out-of-plane bending vibrations) and at 783 cm^−1^ for aromatic C–H bonds) was observed, thus demonstrating the successful incorporation of CQ into the mats (Figure 3c). In the spectrum of the mat, obtained by simultaneous electrospinning of a PHB solution and electrospraying of a PVP,CQ solution, a shift of the band for C=O stretching vibrations of PVP from 1663 cm^−1^ (for PVP*in*PHB mat, Figure 3b) to 1667 cm^−1^ (Figure 3d) was registered. This might be explained by interactions between PVP and CQ. The appearance of new bands for C=C stretching vibrations of CQ at 1576 cm^−1^ and 1489 cm^−1^, as well as two bands at 808 cm^−1^ (for γ_Ar-H_, out-of-plane bending vibrations) and at 783 cm^−1^ (for aromatic C–H bonds) was also observed, which was an indication of the incorporation of CQ in the particles on the PHB mat surface.

**Figure 4:**
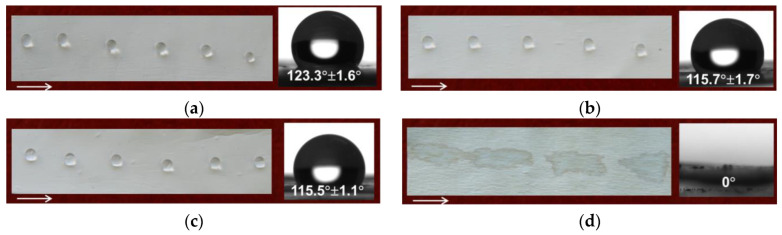
Images of water droplets deposited on the surfaces of mats from: (**a**) PHB; (**b**) PVP*in*PHB; (**c**) PVP,CQ*in*PHB; and (**d**) PVP,CQ*on*PHB. The direction of the collector rotation is demonstrated by an arrow.

The hydrophilic/hydrophobic characteristics of fibrous mats can greatly influence the adhesion and growth of pathogenic fungi in plants. It was of interest to measure the water contact angle of the obtained fibrous materials. We have found that the PHB mat was hydrophobic (water contact angle of 123.3° ± 1.6°) (Figure 4a). The water contact angle value of the PHB fibrous material determined experimentally by us was in good agreement with those reported in the literature values by other authors [46]. The incorporation of 10 wt.% water-soluble polymer PVP resulted in a slight decrease in the hydrophobicity of the mats (the value of the water contact angle was 115.7° ± 1.7°, Figure 4b). The presence of CQ in the PVP,CQ*in*PHB fibers did not result in a change in the water contact angle values (Figure 4c). Moreover, we have find out that the mats fabricated by electrospinning of PHB solution in conjunction with electrospraying of PVP,CQ were hydrophilic. Their water contact angles were 0° (Figure 4d).

**Figure 5:**
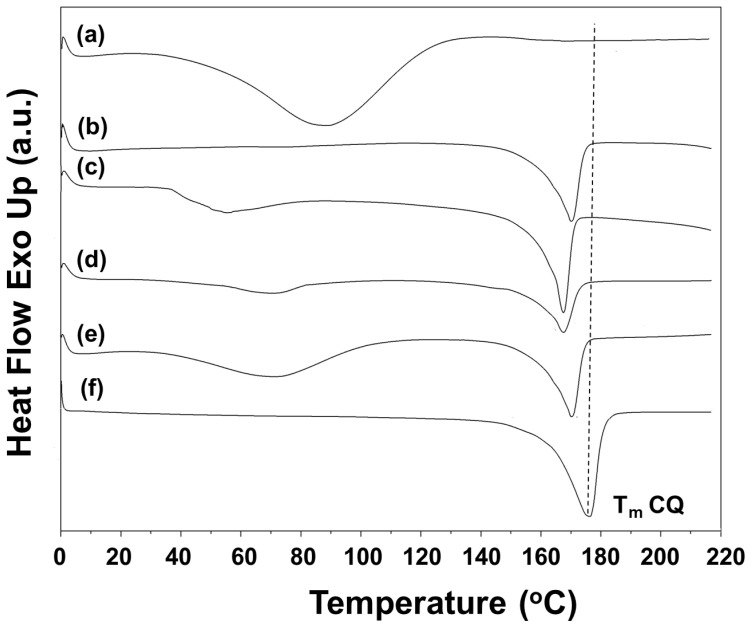
DSC thermograms (first heating run) of (**a**) PVP powder; (**b**) PHB mat; (**c**) PVP*in*PHB mat; (**d**) PVP,CQ*in*PHB mat; (**e**) PVP,CQ*on*PHB mat; and (**f**) CQ powder.

The thermal behavior of the PVP,CQ*in*PHB and PVP,CQ*on*PHB mats was studied by DSC analyses (Figure 5). An endothermic peak at 170 °C; for T_m_ of PHB was detected in the thermogram of the PHB fibers (Figure 5b). Furthermore, in the thermograms of the PVP,CQ*in*PHB mats a 3 cm^−1^ shift of the T_m_ for PHB towards a lower temperature to 167 °C was observed (Figure 5d). The crystallinity degree of PHB in PVP,CQ*in*PHB mats (37%) did not change significantly compared to that of the PVP*in*PHB mats (42%). It can be assumed that when CQ is incorporated in the bulk of the PVP*in*PHB mat, interactions occur between PHB, PVP and CQ, resulting in the diminishing of T_m_ of PHB. An endothermic peak at 170 °C ascribed to PHB melting was registered in the thermograms of the mats obtained by electrospinning in conjunction with electrospraying (Figure 5e). The crystallinity degree of PHB in PVP,CQ*on*PHB mats was 42%, a value that coincides with the crystallinity degree of the PHB fibers (42%). In the cases of PVP powder (Figure 5a), and of fibrous mats containing PVP in the bulk of the mat or on its surface, a broad endothermic peak was detected between 25 °C and 100 °C, due to loss of moisture (Figure 5c–e). Moreover, in the thermograms of the PVP,CQ*in*PHB mats no peak at 176 °C corresponding to CQ melting was observed (Figure 5d). This demonstrated that CQ incorporated in the fibers was in the amorphous state. It can be seen that CQ incorporated in the particles deposited on the PHB fibers was also in the amorphous state (Figure 5e).

**Figure 6:**
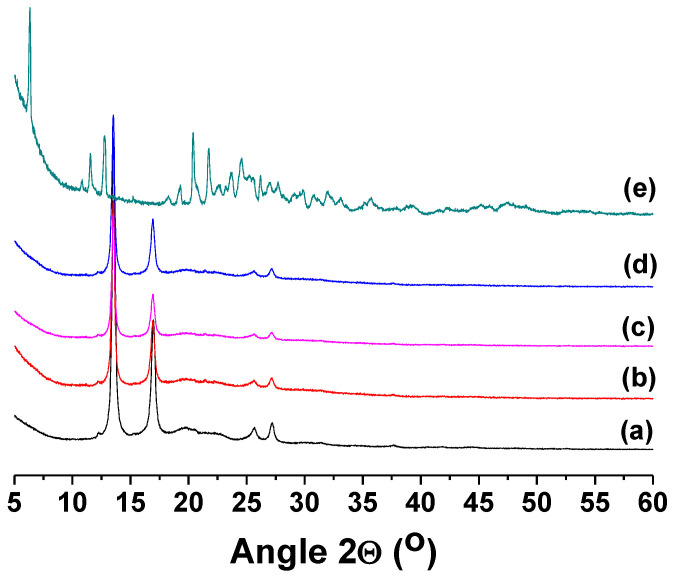
XRD patterns of: (**a**) PHB mat; (**b**) PVP*in*PHB mat; (**c**) PVP,CQ*in*PHB mat; (**d**) PVP,CQ*on*PHB mat; and (**e**) CQ powder.

It is known that crystallinity affects the release of biologically active compounds. The crystallinity of the fabricated fibrous materials was studied by XRD analysis. Figure 6 shows the XRD patterns of PHB, PVP*in*PHB, PVP,CQ*in*PHB, PVP,CQ*on*PHB mats and of CQ powder. In XRD graph of the PHB and PVP*in*PHB fibrous materials only diffractions due to the crystalline phase of PHB (2θ = 13.6°, 17.0°, 20.1°, 22.1°, 25.6° and 27.2°) were registered. In the cases of PVP,CQ*in*PHB and PVP,CQ*on*PHB mats (Figure 6c,d) the main diffractions attributed to the crystalline phase of CQ were not observed (2θ = 6.3°, 12.8°, 20.4°, 21.7° and 24.6°), thus demonstrating that CQ loaded into the mats or in the PVP particles on the PHB fibers was in the amorphous state. Thus, the obtained results were in conformity with the results obtained by the DSC analyses.

In order to elucidate the effect of PVP on the crystallinity of CQ in CQ-loaded PVP*in*PHB fibrous materials, the XRD patterns of CQ*in*PHB mats were also recorded. As seen in Figure S2 (Supporting Materials) in the case of CQ*in*PHB mats, in addition to the diffractions due to the crystalline phase of PHB, the evidence of diffractions corresponding to the crystalline phase of CQ was registered as well. This indicated that, in this case, CQ was in the crystalline state. Therefore, the findings showing the presence of CQ in CQ-containing PVP*in*PHB mats (both “*in*” and “*on*” types) in the amorphous state are consistent with the literature data on diminishing the formation of crystals from drugs interacting with PVP [43,47].

**Figure 7:**
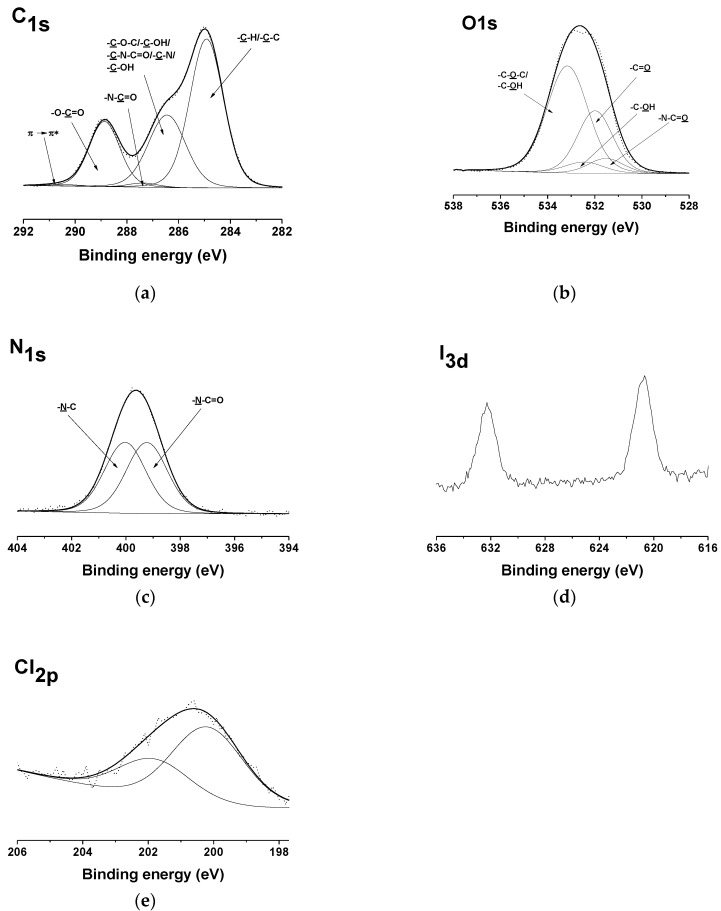
XPS peak fittings for: PVP,CQ*in*PHB mat: [(**a**) C_1s_, (**b**) O_1s_, (**c**) N_1s_, (**d**) I_3d_, (**e**) Cl_2p_].

The successful incorporation of CQ into the PVP*in*PHB mat surface or into the PVP particles deposited on the PHB mat surface was also confirmed by XPS analyses (Figure 7 and Supplementary Material, Figure S3). The appearance of N_1s_ peaks in the spectra of the PVP,CQ*in*PHB mats was observed at 399.2 eV due to -N-C=O from PVP and at 400.0 eV characteristic of -N-C from CQ [48] (Figure 7c). Moreover, the spectra displayed the appearance of an I_3d_ peak—at 620.7 eV (I_3d5/2_) and at 632.2 eV (I_3d3/2_), attributed to the presence of CQ in the mat surface (Figure 7d). Cl_2p_ (at 201.8 eV (Cl_2p1/2_) and at 200.2 eV (Cl_2p3/2_) (Figure 7e) peaks were also detected confirming the incorporation of CQ in the PVP,CQ*in*PHB mat surface. Five peaks were detected in the detailed C_1s_ spectrum of the PVP,CQ*in*PHB mat (Figure 7a). The signal at 285 eV was ascribed to -C-H or -C-C- from PHB, PVP and from CQ, and that at 286.5 eV was attributed to -C-O-C, -C-OH from PHB, to -C-N-C=O from PVP and also to -C-N and -C-OH from CQ. The peak at 287.4 eV was corresponding to -N-C=O from PVP and at 288.9 eV to -O-C=O from PHB. The presence of a peak at 290.4 eV for the π→π* shake-up satellite due to the aromatic ring of the incorporated CQ was registered. Four components demonstrated the detailed O_1s_ spectrum—at 531.5 eV ascribed to -N-C=O from PVP, at 532.0 eV to -C=O from PHB, at 532.5 eV to -C-OH from CQ and at 533.2 to -C-OH and -C-O from PHB (Figure 7b). The presence of the detected peaks are in accordance with the structure of the PVP,CQ*in*PHB fibrous material.

The theoretical ratio of the peak area for the respective carbon atoms was [C-C/C-H]/[C-O/C-OH/C-N-C=O/C-N/C-OH]/[N-C=O]/[O-C=O]/[π→π*] = 50.3/27.2/1.3/20.2/1.0, while the experimental ratio was 50.6/27.4/1.2/20.2/0.6. Therefore, the largest area was registered for the peak of the carbon atoms engaged in the C-C/C-H bonds. The obtained results were in conformity with the hydrophobicity of the surface of the PVP,CQ*in*PHB fibrous material. The value of the water contact angle was 115.5° ± 1.1° (Figure 4c).

Significant differences in the expanded C_1s_ spectrum of the PVP,CQ*on*PHB mat were registered in comparison to the C_1s_ spectrum of the PHB mat (Supplementary Material, Figures S3a and S4a). Supplementary Material, Figure S3a presented two new peaks at 287.3 eV ascribed to -N-C=O from PVP and at 290.4 eV to the π→π* shake-up satellite of the CQ aromatic ring. There was also an increase in the intensity of the peak at 286.4 eV ascribed to -C-O-C and -C-OH from PHB, to -C-N-C=O from PVP, as well as to -C-N and -C-OH from CQ. In the detailed O_1s_ spectrum of PVP,CQ*on*PHB fibrous material, the presence of two new peaks was identified—at 531.6 eV, corresponding to -N-C=O from PVP and at 532.6 eV, ascribed to -C-OH from CQ (Supplementary Material, Figure S3b). The comparison of the detailed N_1s_ spectrum of these fibrous materials with that of the PHB mats revealed the presence of two new components—at 399.1 eV characteristic for -N-C=O from PVP and at 400.0 eV due to -N-C from CQ (Supplementary Material, Figure S3c). The presence of peaks for N_1s_, I_3d_ (at 620.7 eV (I_3d5/2_) and at 632.2 eV (I_3d3/2_)) and Cl_2p_ (at 201.6 eV (Cl_2p1/2_) and at 200.0 eV (Cl_2p3/2_)) (Supplementary Material, Figure S3c–e) confirmed the incorporation of CQ into PVP particles deposited on the surface of PHB mat.

### 3.2. In Vitro CQ Release Studies

**Figure 8:**
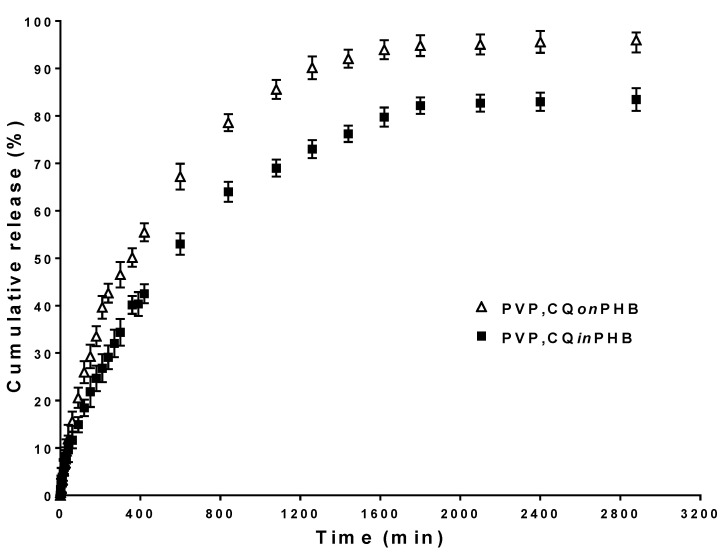
In vitro study of the CQ release from: PVP,CQ*in*PHB and PVP,CQ*on*PHB mats in acetate buffer/Tween 80 (99/1 *v*/*v*) at 25 °C, pH 3.6, ionic strength 0.1.

The in vitro study of the CQ release from PVP,CQ*in*PHB and PVP,CQ*on*PHB mats was assessed spectrophotometrically in acetate buffer (pH 3.6) containing Tween 80 (99/1 *v*/*v*), for 48 h at 25 °C. These fibrous materials containing the water-soluble polymer PVP showed rapid initial release with subsequent gradual release profile (Figure 8).

As seen from Figure 8, CQ was released faster and in a greater amount when incorporated into PVP particles that are deposited on the PHB fiber surface than when incorporated in the PVP*in*PHB fibers. About 78.6% and 64% of the loaded CQ was released in the initial 840 min in the case of PVP,CQ*on*PHB and PVP,CQ*in*PHB mats, respectively (Figure 8). The amount of CQ released from the PVP,CQ*in*PHB mats for 2880 min was ca. 83.5%. For PVP,CQ*on*PHB fibrous materials the total amount of CQ released in 2880 min was 96%. This result might be due to the difference in the diffusion of CQ incorporated in the bulk of the fibers and the diffusion of CQ through the PVP particles deposited on the fiber surface. The obtained results from the CQ release studies showed that the CQ release from the fibrous materials was assisted by the presence of PVP in the fibrous materials or on their surface. These results are consistent with our previous findings on an increase in the rate of release of 8-hydroxyquinoline derivatives from other fibrous systems upon incorporation of a water-soluble polymer [21,22]. In the present study, using one-pot electrospinning or electrospinning in conjunction with electrospraying, fibrous materials of diverse design and with different CQ release profiles were fabricated. The obtained results show that the CQ-containing materials are perspective candidates for application in agriculture as active dressings for grapevine protection from fungal pathogens.

### 3.3. Antifungal Assay

8-hydroxyquinoline derivatives are known for their good antibacterial and antifungal properties [49]. Among the 8-hydroxyquinoline derivatives, CQ manifested the ability to inhibit the growth of a large number of fungi [24,25].

Until now, no data on the antifungal activity of CQ, as well as of fibrous materials containing this biologically active compound against *P. chlamydospora* and *P. aleophilum*, which are the main fungal species causing esca disease, have been reported.

**Figure 9:**
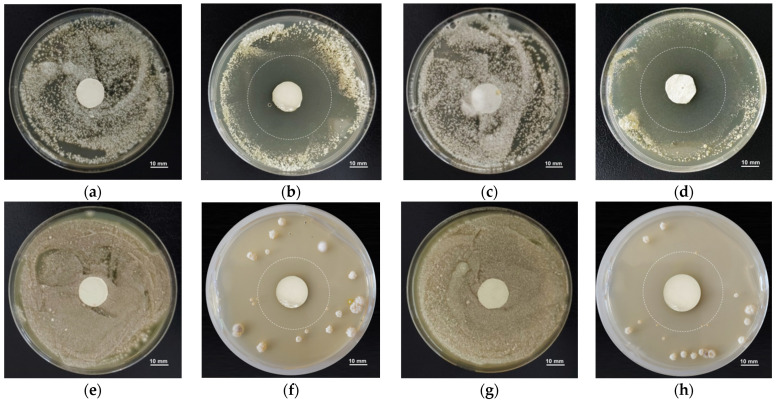
Digital images of the zones of inhibition against *P. chlamydospora* (**a**–**d**) and *P. aleophilum* (**e**–**h**) after the contact of the fibrous materials with fungi cells: (**a**,**e**)-PVP*in*PHB mat; (**b**,**f**)-PVP,CQ*in*PHB mat; (**c**,**g**)-PHB mat; and (**d**,**h**)-PVP,CQ*on*PHB mat; scale bar = 10 mm.

Therefore, we have studied the antifungal activity of fibrous materials loaded with CQ by performing microbiological assays against the fungi *P. chlamydospora* and *P. aleophilum*. The diameters of the inhibition zone around the fibrous discs and MIC values for CQ against the two used fungi were determined as well. The MIC values were 10 and 1 μg/mL, respectively. The growth of *P. chlamydospora* and *P. aleophilum* was studied for the time period of 96 h. As can be seen from Figure 9a,c,e,g, the PHB and PVP*in*PHB mats did not show any significant antifungal effect.

In contrast, CQ-containing mats exhibited antifungal activity against these fungi and well-defined zones of inhibition of fungal cell growth were detected (Figure 9b,d,f,h). These well-defined zones illustrated that the release profile of CQ provided a sufficient amount of the biologically active compound even in the initial experiment stages. The values of the mean diameter of the inhibition zones for PVP,CQ*in*PHB and PVP,CQ*on*PHB mats for the tests against *P. chlamydospora* did not differ significantly: 44.2 ± 1.1 mm and 45.0 ± 1.3 mm, respectively (Figure 9b,d). For the tests against *P. aleophilum*, the diameters of the zones of inhibition around PVP,CQ*in*PHB and PVP,CQ*on*PHB fibrous materials were 36.7 ± 1.9 and 41.2 ± 3.0 cm, respectively (Figure 9f,h). The obtained results indicated that the incorporated CQ imparted good antifungal activity against species *P. chlamydospora* and *P. aleophilum* to the mats.

The antifungal activity of CQ against *Candida spp.* and dermatophytes was suggested to be due mainly to damage of the cell wall, resulting in the death of fungal cells [50]. However, up to now the mechanism of action of CQ in fungal cells has not been fully clarified. There are no data in the literature on the mechanism of action of CQ against fungal cells of *P. chlamydospora* and *P. aleophilum*. We hypothesize that the observed antifungal activity of CQ-containing fibrous materials against the fungi *P. chlamydospora* and *P. aleophilum* is most likely due to their damaging effect on the fungal cell wall.

## 4. Conclusions

Novel fibrous materials containing CQ of different design were fabricated by electrospinning (“*in*” strategy) or by electrospinning in conjunction with electrospraying (“*on*” strategy). It was demonstrated that the presence of PVP capable of forming hydrogen bonds with CQ facilitated the release of CQ from the mats. The release of CQ from PVP,CQ*on*PHB fibrous materials was faster than the release from the PVP,CQ*in*PHB fibrous materials. We found that CQ was an agent with good efficacy against the fungal pathogens *P. chlamydospora* and *P. aleophilum* (MIC were 10 μg/mL and 1 μg/mL, respectively). Furthermore, CQ-containing fibrous materials (both “*in*” and “*on*” types) exhibited significant antifungal activity against *P. chlamydospora* and *P. aleophilum*. All these results clearly reveal that the prepared fibrous materials are promising as active dressings for the protection of grapevine against two main esca-causing fungal pathogens.

## Supplementary Materials

The following are available online at https://www.mdpi.com/article/10.3390/polym14030367/s1, Figure S1: ATR-FTIR spectra of PHB mat, Figure S2: XRD patterns of PHB mat, CQ and CQ*in*PHB mat, Figure S3: XPS peak fittings for PVP,CQ*on*PHB mat, Figure S4: XPS peak fittings for PHB mat.
