# Supplementary material for: Electrospun 5-Chloro-7-iodo-8-hydroxyquinoline (Clioquinol)-Containing Poly(3-hydroxybutyrate)/Polyvinylpyrrolidone Antifungal Materials Prospective as Active Dressings against Esca
Source: Polymers (Basel). 2022 Jan 18;14(3):367. doi: 10.3390/polym14030367 (PMC8838117; doi:10.3390/polym14030367)
Supplement: Supplementary file 1 [file polymers-14-00367-s001.zip › polymers-1554566-supplementary.pdf]

Electronic Supplementary Material

Electrospun 5-Chloro-7-iodo-8-hydroxyquinoline (Clioquinol)-Containing Poly(3-hydroxybutyrate)/Polyvinylpyrrolidone Antifungal Materials Prospective as Active Dressings Against Esca

Milena Ignatova[1,*], Nasko Nachev[1], Mariya Spasova[1,*], Nevena Manolova[1], Iliya Rashkov[1] and Mladen Naydenov[2]

[1]*Laboratory of Bioactive Polymers, Institute of Polymers, Bulgarian Academy of Sciences, Acad. G. Bonchev St, Bl. 103A, BG-1113 Sofia, Bulgaria; nachev_n@ polymer.bas.bg (N.N.); manolova@polymer.bas.bg (N.M.); rashkov@polymer.bas.bg (I.R.)*

[2]*Department of Microbiology, Agricultural University, BG-4000 Plovdiv, Bulgaria; mladen@au-plovdiv.bg (M.N.)*

*Correspondence: mspasova@polymer.bas.bg (M. S.), ignatova@polymer.bas.bg (M. I.); Tel.: +359 (0)2 9793468; Fax: +359 (0)2 8700309

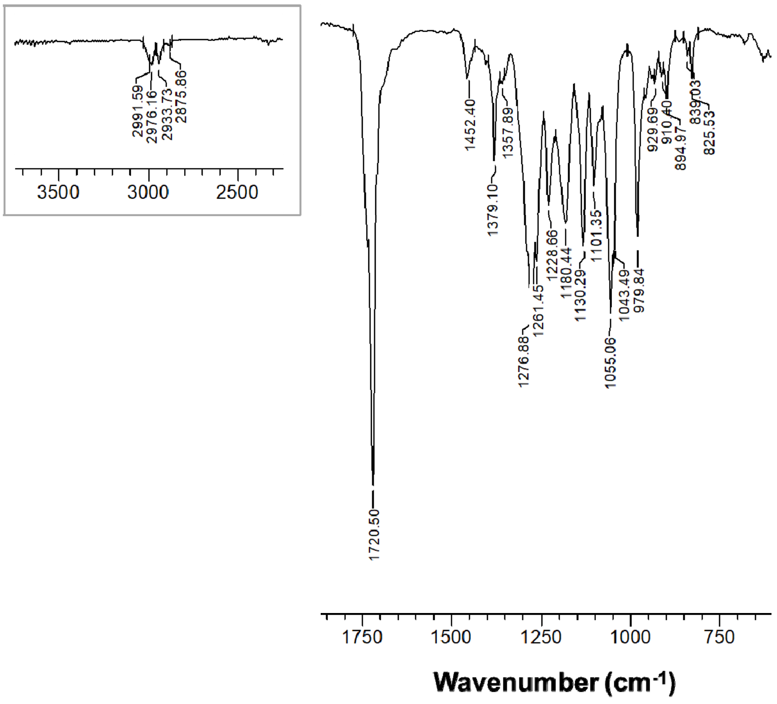

Figure S1. ATR-FTIR spectra of PHB mat, in the range from 1750 to 750 cm^{-1} and from 3500 to 2500 cm^{-1} (inset).

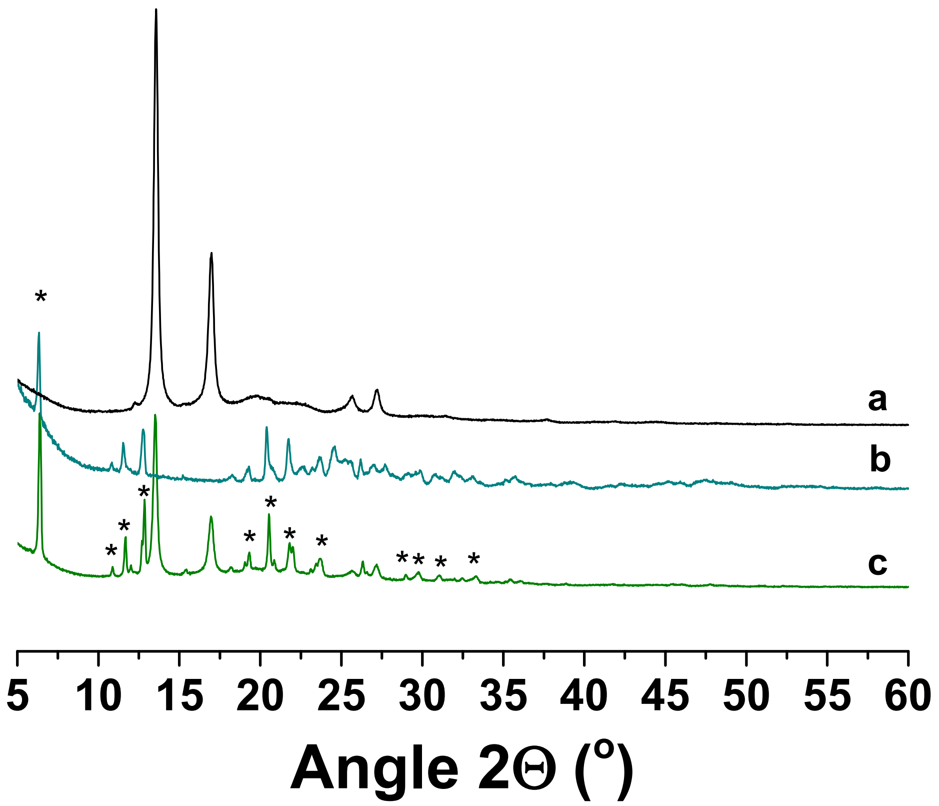

Figure S2. XRD patterns of: (a) PHB mat, (b) CQ and (c) CQ*in*PHB mat. The diffraction peaks for crystal phase of CQ incorporated in the bulk of PHB fibers are denoted with asterisk.

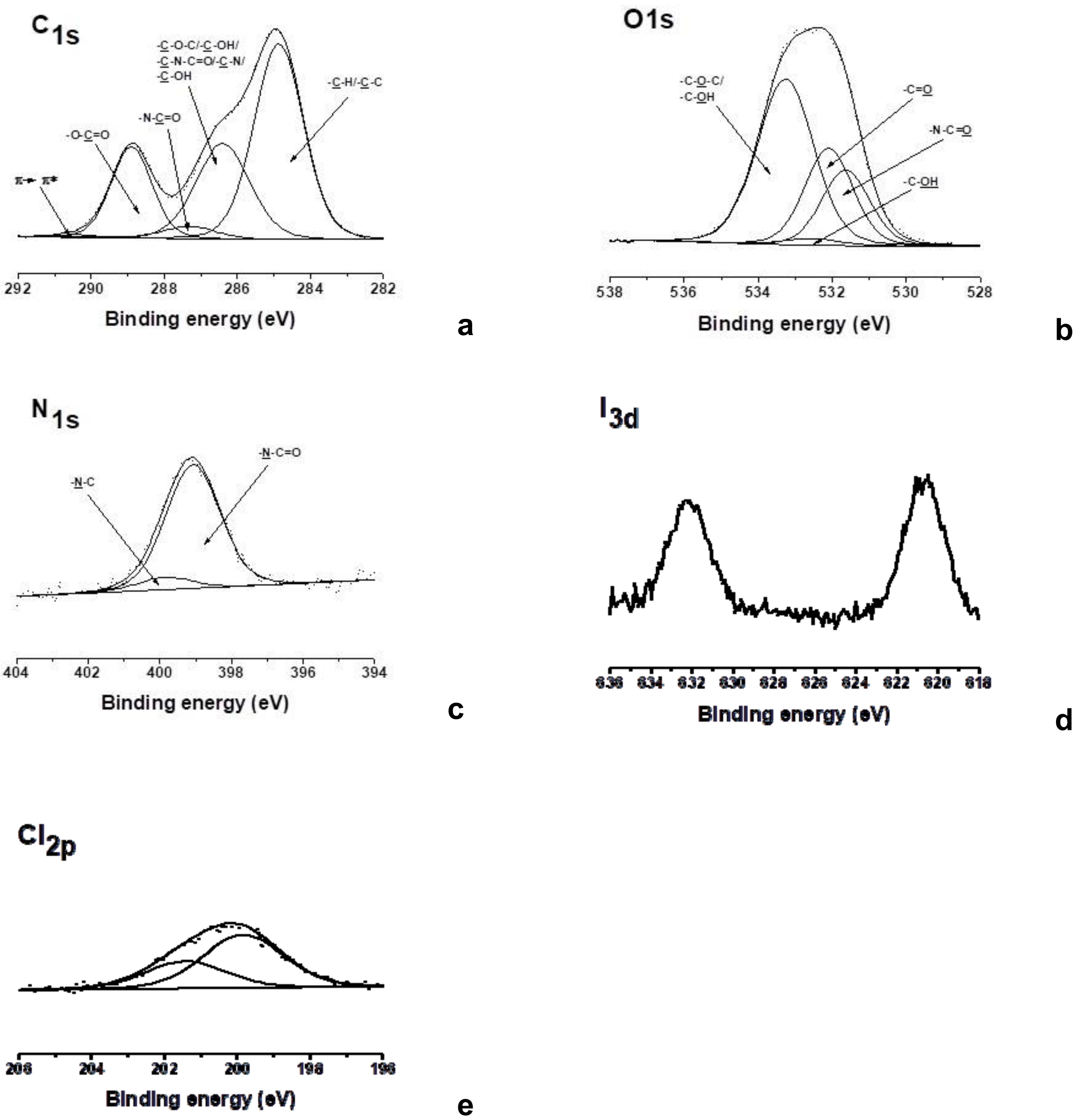

Figure S3. XPS peak fittings for PVP,CQ*on*PHB mat [(a) C_{1s}, (b) O_{1s}, (c) N_{1s}, (d) I_{3d}, (e) Cl_{2p}].

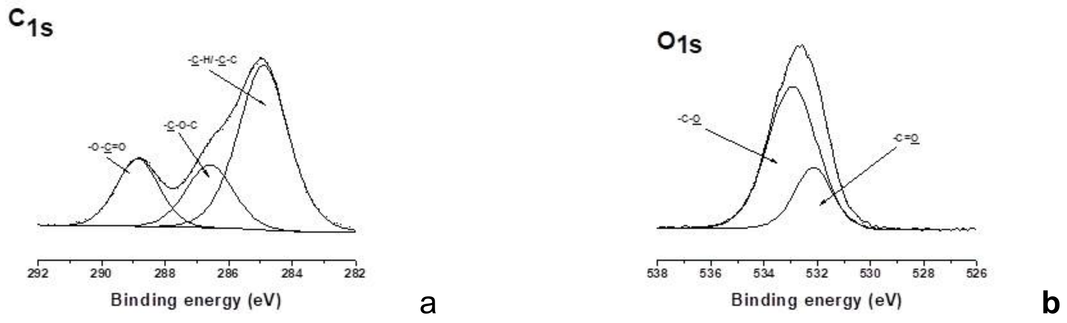

Figure S4. XPS peak fittings for PHB mat [(a) C_{1s}, (b) O_{1s}].
